# A systematic review on gut–brain axis aberrations in bipolar disorder and methods of balancing the gut microbiota

**DOI:** 10.1002/brb3.3037

**Published:** 2023-05-01

**Authors:** Crystal Obi‐Azuike, Ruona Ebiai, Taneil Gibson, Ariana Hernandez, Asma Khan, Gibson Anugwom, Alexsandra Urhi, Sakshi Prasad, Sara Ait Souabni, Funso Oladunjoye

**Affiliations:** ^1^ Department of Psychiatry and Behavioral Sciences Tulane University School of Medicine New Orleans, Los Angeles USA; ^2^ Department of Internal Medicine Ochsner Clinic Foundation New Orleans Louisiana USA; ^3^ Department of Psychiatry University of Medicine and Health Sciences (UMHS) New York New York USA; ^4^ Department of Psychiatry Lake Erie College of Osteopathic Medicine Erie Pennsylvania USA; ^5^ Menninger Department of Psychiatry & Behavioral Sciences Baylor College of Medicine Houston Texas USA; ^6^ Department of Mental Health Federal Medical Center Asaba, Delta State Nigeria; ^7^ Department of Psychiatry National Pirogov Memorial Medical University Vinnytsya, Vinnytsya Ukraine; ^8^ Faculty of Medicine and Pharmacy of Marrakesh Cadi Ayyad University ‐ Marrakech, Marrakech Morocco

**Keywords:** biomarker, bipolar depression, bipolar disorder, gut microbiome, gut microbiota, gut microbiota, mania, mood disorder, probiotic

## Abstract

**Background:**

Bipolar disorder (BD) is a mood disorder that affects millions worldwide. Up to half of the diagnosed patients are reported to not receive adequate treatment. This study aims to assess the relationship between the gut–brain axis and BD and to discuss and compare the efficacy of varying methods of balancing gut microbiotas in BD.

**Methods:**

Using PubMed, Embase, and Google Scholar from November 2021 to February 2022, we found 5310 studies on gut microbiota and its relation to BD. Using our inclusion criteria, 5283 studies were excluded. A total of 27 full‐text articles were assessed for eligibility. Also, 12 articles that met our criteria and eligibility criteria reported on 613 BD patients.

**Results:**

Most studies analyzed found an overall difference in gut microbiota composition in bipolar patients compared to healthy controls, though the alterations found were not consistent. Differences in *Lactobacillus, Faecalibacterium*, and *Ruminococcus* abundance in BD compared to controls were found to be the most consistent across a few of the studies, but their effects on the gut–brain axis conflicted. Probiotic supplementation was found to lower patient rehospitalizations and significantly improve depressive symptoms and cognitive impairments among patients with BD.

**Conclusions:**

Multiple studies included in this review point toward a possible link between BD and the gut microbiota. Probiotic supplements and other gut‐balancing therapies could serve as effective adjunctive methods for the treatment of BD. Notable limitations of the studies included for analysis were small sample sizes and majority observational study designs. Furthermore, the microbiota aberrations found in patients with BD were not consistent across multiple studies. Despite these limitations, our findings demonstrate the need for further research regarding the relationship between aberrant gut microbiota profiles and BD, as well as the effectiveness of gut balancing methods as adjunctive treatments.

## Graphical abstract

In this systematic review, the link between the gut microbe and the development and progression of bipolar disorder is investigated. Furthermore, the potential use of gut balancing methods such as probiotics as adjunctive therapy for bipolar symptoms is analyzed. Our investigation found a link between gut microbiota composition and bipolar disorder, as well as evidence that supports the use of probiotics as an adjunctive treatment.

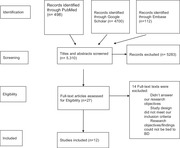

## 1 INTRODUCTION

Bipolar Disorder (BD) affects 45 million people worldwide (World Health Organization, 2022). The National Institute of Mental Health has reported that up to 50% of patients with BD fail to receive adequate treatment for their mental illness, leaving over 2 million US patients untreated (Bipolar Disorder‐ Fact Sheet, 2022). Globally, it has been reported by the World Health Organization (2022) that 75% of people with mental health disorders, such as BD, lack access to adequate treatment or care. It has also been found that even with adequate treatment, 37% of patients will experience a breakthrough affective episode within a year, and 60% will experience a relapse of affective symptoms in 2 years (Da Costa et al., 2016). Bipolar symptom relapse and worsening disease progression has become a growing concern, as studies have found that a significant proportion of patients are experiencing symptom relapse despite the use of adequate pharmacological agents (Fountoulakis, 2012; Geddes & Miklowitz, 2013; Perlis et al., 2006). These observed trends highlight the need for further research into possible adjunctive methods of treatment for BD.

The neurobiological mechanisms that drive BD symptoms are poorly understood, which could be a possible contributor for increasing rates of BD symptom relapse (Aizawa et al., 2019; Geddes & Miklowitz, 2013; Perlis et al., 2006). A possible environmental factor that has yet to be adequately studied in reference to BD is the contribution of intestinal microbiota aberrations. An emerging concept of a microbiota–gut–brain axis is being utilized to highlight the significant effect gut microbiota composition has on bidirectional gut–brain communication pathways (Cryan & Dinan, 2012; Painold et al., 2019). Research is revealing that the gut microbiota is key for maintaining homeostasis, and alterations in its composition can lead to a number of disease states including those of the central nervous system (CNS) (Cryan & Dinan, 2012). In fact, the relationship between gut microbiota dysbiosis and host illness has long been linked to chronic conditions such as metabolic syndrome, irritable bowel syndrome, inflammatory bowel disease, and anorexia nervosa. (Cryan & Dinan, 2012; Mayer, 2011; Painold et al., 2019). Gut microbiota alterations have also been linked to other psychiatric disorders including BD (Coello et al., 2019; Hu et al., 2019; Painold et al., 2019). Recurring effective episodes in BD are associated with a progressive decline in cognitive and executive function, and there is growing evidence that supports a relationship between cognition and microbiota via the gut–brain axis (Cryan & Dinan, 2012; Forsythe et al., 2010; Misiak et al., 2020). Thus, the link between BD and the gut microbiome may be a useful treatment alternative, as mounting research has found significant intestinal microbiota alterations in patients with mood disorders (Aizawa et al., 2019; Coello et al., 2019; Forsythe et al., 2010; Hu et al., 2019; Painold et al., 2019).

Disruptions to the neural‐gut pathway have shown a clear link to alterations in the physiological stress response and behavior (Cryan & Dinan, 2012; Forsythe et al., 2010), and the high rates of co‐occurring stress related to psychiatric conditions among patients with gastrointestinal (GI) disorders highlights the gut–brain axis’ role in pathophysiology (Cryan & Dinan, 2012; Mayer, 2011). Research has found that stress, including social disruption, influences gut microbiota composition, and the bidirectional communication between the gut and the CNS plays a role in stress reactivity (Bailey et al., 2011; Painold et al., 2019). The body's stress response includes immune modulation (such as cytokine release), which has been linked to the development of anxiety and depression (Bailey et al., 2011). There is also evidence of a link between stress exposure and the GI barrier; increases in stress increase gut permeability (Misiak et al., 2020). The blood‐brain‐barrier (BBB)’s integrity depends on the intestinal microbiota, as alterations in their composition could lead to BBB impairment (Misiak et al., 2020).

The major phyla of the gut microbiome include *Firmicutes, Bacteroidetes, Actinobacteria, Proteobacteria, Fusobacteria, and Verrucomicrobia*, and two phyla, *Firmicutes* and *Bacteroidetes*, which represent 90% of the gut microbiota (Rinninella et al., 2019). The *Firmicutes* phylum is made up of over 200 genera, including *Bacillus, Lactobacillus, Clostridium, Ruminicoccus*, and *Enterococcus*, of which *Clostridium* constitutes 95% (Rinninella et al., 2019). Bacteroidetes major genera are *Bacteroides and Prevotella* (Rinninella et al., 2019). Actinobacteria is the least abundant phylum and is mainly comprised of *Bifidobacterium* (Rinninella et al., 2019). The gut microbiota composition is thought to vary in the same individual as well as between different individuals due to factors such as age, environmental factors, metabolic factors, and antibiotic use (Rinninella et al., 2019). A few studies have investigated the specific microbial gut composition changes that occur with stress. An animal study found that there was a decrease in *Bacteroides* species (spp.) abundance as well as an increase in *Clostridium* abundance among stress‐exposed mice as compared to controls (Bailey et al., 2011). Chronic psychological stress has been associated with an increase in *Enterobacteriaceae, Escherichia coli*, and a decrease in *Lactobacilli* spp. (Rinninella et al., 2019).

As initially discussed, chronic stress has been found to influence gut microbiota composition through proinflammatory cytokines, such as interleukin‐6 (IL‐6), which was found to be correlated with higher levels of *Lactobacillus*, *Bacilli*, and *Streptococcaceae* (Painold et al., 2019). As stress induces inflammation, biological indices have confirmed inflammation as a major contributor to the pathogenesis of BD. Additionally, increased levels of cortisol in response to stress have been linked to the manic phase of BD (Misiak et al., 2020; Van Den Berg et al., 2020; Τournikioti et al., 2018), including a study that found that cortisol levels were increased for months prior to a manic relapse. This appears to coincide with research that found a significant association between cortisol and ACTH with BD. A notable finding is the relationship between HPA axis dysfunction (which was linked to developmental stressors including childhood trauma), and the clinical presentation of BD (Belvederi Murri et al., 2016).

The shifts in the microbiome composition observed in patients with BD could be of notable significance, and addressing them may contribute to symptom resolution. In this systematic review, we aim to assess how components of the gut–brain axis contribute to BD, to determine how balancing gut microbiotas affect the severity of symptoms, and to discuss and compare the efficacy of varying methods of balancing gut microbiotas in BD.

## 2 MATERIALS AND METHODS

### 2.1 Search strategy

For this systematic review, we searched PubMed, Google Scholar, and Embase for articles published between April 2017 and January 2021. Search terms on PubMed were (gut microbiotas mood disorders) AND (probiotic treatment BD) AND (microbiotas BD) AND (treatment target gut microbiome mood) AND (gut biomarkers mania) AND (gut biomarkers mania) AND (treatment target gut microbiome mood disorders). Search terms on Google Scholar were ((probiotic treatment BD) AND (gut microbiota changes in bipolar). Search terms on Embase were “gut microbiotas mood disorders” OR ((“gut”/exp OR gut) AND (“microbiotas”/exp OR microbiotas) AND (“mood”/exp OR mood) AND (“disorders”/exp OR disorders)) AND (probiotics) AND (mania) AND (gut) AND (biomarkers) AND (bipolar) AND (gut microbiota changes in bipolar) AND (gut biomarkers mania)).

All authors initially selected articles via manual screening of abstracts and searched the reference lists of the chosen articles for additional information that could be applied to our investigation. Articles that investigated the gut microbiome and its link to mood disorders were included.

### 2.2 Study selection

Studies were selected according to the following criteria: population, outcome(s) of interest or condition, study design, and context:

- Population: Patients with diagnosed BD.
- Outcome(s) of interest or condition: The first outcome was determining an association between the gut–brain axis and BD. The second outcome was to document any noted changes in symptom severity of patients after correcting the gut microbiota in affected patients. And last, to evaluate existing methods of gut microbiota balancing in bipolar patients.
- Study design and context: Eligible studies were randomized controlled trials, double‐blind controlled trials, cohort studies, cross‐sectional studies, and case‐control studies.

#### 2.2.1 Inclusion criteria

Human studies that examined and reported (a) a link between gut microbiotas and the physiologic causes of BD (e.g., manic, hypomanic, or depressive symptoms), (b) current documented changes to gut microbiota using probiotics, supplements, fecal implantation, or diet, (c) any functional, depressive, and/or manic symptom changes observed with gut microbiota balancing.

#### 2.2.2 Exclusion criteria

Systematic review studies, editorials, case studies, commentaries, and articles irrelevant to either the development or treatment of BD (e.g., manic, hypomanic, or depressive symptoms) or the behavioral changes associated with gut microbiota.

### 2.3 Data collection & study assessment

All authors independently reviewed the abstracts of all the articles identified. We divided the articles into two groups of “Adopted” and “Not Adopted” based on the inclusion criteria. Then, we screened the “Adopted” articles and created a spreadsheet to include them to be used for our research work. After the final selection process, a Preferred Reporting Items For Systematic Reviews and Meta‐Analysis (PRISMA) flow chart was generated following the PRISMA guidelines. Resources for this review were obtained via qualitative and quantitative analysis.

### 2.4 Data synthesis & analysis

Quantitative and qualitative studies based on original research that examined the gut microbiota and brain relationship in BD and nonpharmacologic methods for treating bipolar symptoms were included. The data synthesis was conducted in a detailed summary of the included studies by table construction. The quantitative data were extracted using Microsoft Word. The data were grouped according to the objective of this study.

## 3 RESULTS

**FIGURE 1:**
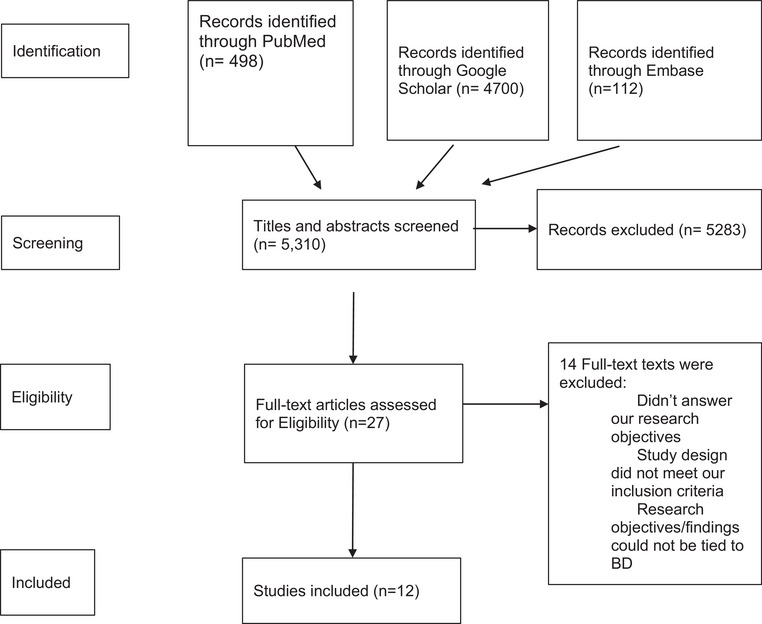
Preferred reporting items for systematic reviews and meta‐analyses (PRISMA) study selection flow chart.

Our study included 613 BD patients (mean age: 39.25 y, women: 58%), 39 first‐degree relatives, and 321 healthy controls (mean age: 36.4 y, women: 57%). Other characteristics such as BMI, medication status, lifestyle modifications, and BD I vs. BD II diagnosis were collected during this analysis and are listed in Table 1. The study selection flow chart is given in Figure 1.

**TABLE 1:** Summary of the studies included in our review.

**Studies included**	**Type of study**	**Study participant characteristics**	**Gut–brain axis changes in BD**	**BD symptom changes after gut microbiota alterations**	**Effectiveness of gut microbiota balancing methods for BD**	**Summary of findings**
**Interventional studies**						
Eslami Shahrbabaki et al. (2020)	Randomized, Double‐Blind, Placebo‐controlled trial	Type 1 patients with BD‐38 Probiotic: 25 ‐ Mean age: 38.9 y Placebo: 25 ‐ Mean age: 35 y Lost to follow‐up: 12 (6 in each group) *Patients in both groups were allowed to receive lithium oxide, with a maximum dose of 900 mg per day, sodium valproate, with a maximum dose of 1200 mg per day, and, if necessary, risperidone	–	No significant reductions in Young Mania Rating Scale (YMRS) and Hamilton's Depression Rating Scale (HDRS) scores over time between placebo and probiotic groups, but probiotic consumption significantly reduced the severity of depression and mania over time.		There is a decrease in the severity of depression and mania in the group using probiotics, despite no significant changes in depression or mania between the control and placebo groups.
Dickerson et al. (2018)	Randomized controlled trial	Recently hospitalized manic patients: 66 Probiotic group: 33 Placebo group: 33 Mean age probiotic group: 37.9 years Mean age placebo group: 33.3 years Probiotic group females average: 24 years Placebo group females average: 18 years Cigarette smoking probiotic group: 14 Cigarette smoking placebo group: 13 BMI probiotic group: 29.2 BMI placebo group: 31.6 In the Probiotic group: ‐YMRS score: 11.9 ‐Bipolar I, manic:17 ‐Bipolar I, mixed: 8 ‐Schizoaffective disorder bipolar type: 8 ‐Antipsychotics: 43 ‐Mood stabilizers: 20 ‐Lithium: 13 ‐Antidepressants: 8	‐		Variants of *Bifidobacterium* and *Lactobacillus* in probiotic patients led to reductions in hospitalizations. Significant reductions in psychiatric rehospitalization frequency and duration (a total of 182 fewer days rehospitalized during the trial)	There are lower rehospitalization rates in manic patients with supplementation of probiotics as compared to the control group.
**Observational Studies**						
Aizawa et al. (2019)	Case‐control study	**Patients with BD‐39** Females: 22 Males: 17 Mean age: 40.3 y Mean HAM‐D 17: 10.3 Mean YMRS: 2.1 Probiotic use: 9 pts Medication: ‐ Antipsychotic: 13 ‐ Antidepressant: 12 ‐ Sodium Valproate: 8 ‐ Lamotrigine: 13 ‐ Carbamazepine: 4 **Healthy controls‐58** Age: 43.1 y Females: 36 Males: 22	No significant difference was found in either bacterial count between patients with BD and HCs. Low *Bifidobacterium* levels were found in correlation to low cortisol levels, but no significant difference in cortisol levels was found in BD or controls.	–	–	No significant bacterial differences were found between BD and controls. *Lactobacillus* was negatively correlated to sleep. Bifidobacterium was negatively correlated to cortisol levels.
Painold et al. (2019)	Cross‐sectional study	Patients with BD (medicated): 32 Males: 18 Females: 14 Mean age: 41.3 years Mean BMI: 24.6 BDI‐II score: 18 Atypical antipsychotics: 24 Lithium: 8 Anticonvulsants: 11 Antidepressants: 23 Healthy controls‐10 Females: 6 Mean age: 31.4 Mean BMI: 24.26	‐higher IL6 levels had higher levels of *Lactobacillus*, *Streptococcaceae*, and *Bacilli*. *Faecalibacterium* found to be higher in HCs versus patients with BD.	–		Gut microbiota can affect inflammatory markers like IL‐6 through its direct correlation with increased *Lactobacillus*, *Streptococcaceae*, and *Bacilli* levels.
Coello et al. (2019)	Cross‐sectional study	**Patients with BD‐113** ‐ Mean age: 31 years ‐ Females: 70 ‐ Males: 43 ‐ BMI: 24.8 kg/m2 ‐ Waist circumference: 85.5 cm ‐ Mean Physical activity: 1980 MET‐minutes per week ‐ Smokers: 40 ‐ HDRS‐17: 10 ‐ YMRS: 2 ‐ BD I: 44 ‐ BD II: 65 ‐ Illness duration: 11 y ‐ No psychotropic medication: 14 ‐ Lithium treatment: 44 ‐ Antidepressant treatment: 30 ‐ Antipsychotic treatment: 43 **Unaffected first‐degree relatives‐ 39** ‐ Mean age: 28 ‐ Females: 21 ‐ Males: 18 ‐ BMI: 24.4 kg/m2 ‐ Waist circumference: 80 cm ‐ Physical activity: 2400 MET‐minutes per week ‐ Smokers: 10 ‐ HDRS‐17: 2 ‐ YMRS: 0 **Healthy controls‐ 77** ‐ Mean age: 29 ‐ Females: 47 ‐ Males: 30 ‐ Number of smokers: 8 ‐ BMI: 24.2 kg/m2 ‐ Waist circumference: 85.5 cm ‐ Physical activity: 2160 MET‐minutes per week ‐ HDRS‐17: 0 ‐ YMRS: 1	*Flavinofactor* was significantly more prevalent in patients with BD compared to healthy individuals. 90% of all serotonin is produced in GI tract affecting vagus nerve and permeability of BBB barrier.	–	–	Microbiota genus *Flavonifractor* is prevalent in patients with BD and may induce host inflammation.
Hu et al. (2019)	Case‐control study	**Patients with BD**‐52 Mean age: 24.15 Females: 25 Males: 27 Mean MADRS score: 28.15 Mean HDRS‐17: 30.15 Mean YMRS: 1.87 BD I: 12 BD II: 38 NOS: 2 Family History: ‐ Yes: 14 ‐ No: 38 **Healthy controls**‐ 45 Mean age: 36.29 Females: 22 Males: 23	Gut microbiota compositions in untreated patients with BD were dominantly characterized by *Bacteroidetes*. In healthy controls, gut microbiota compositions were dominantly characterized by *Firmicutes*. Microbiotas producing butyrate were abundant in controls and absent in untreated BD patients. Patients with BD had lower levels of bacteria who use SCFA's to reduce inflammation	–	–	Gut microbiota compositions were significantly different in patients with BD. The amounts of specific genera could be correlated with depressive severity. Patients with BD and healthy controls could be distinguished by gut microbiota meaning that microbial markers could be used in treatment.
Evans et al. (2017)	Case‐control study	Patients with BD‐115 and healthy controls (HC)‐64 Mean age HC: 48.6 Mean age BD: 50.2 Females HC: 40 Females BD: 83 Mean BMI HC: 26.0 Mean BMI BD: 29.3 BD 1: 76 BD NOS: 10 BD II: 29	Decreased levels of *Faecalibacteriu*, a gut bacterium associated with a balanced microbiota, in bipolar patients compared to healthy controls	*Faecalibacterium* was associated with improved depression scores (PHQ9), sleep quality scores, and improved physical health. Changes were specifically seen in sleep quality, latency, and changes to daytime lethargy rating via Pittsburg Sleep Quality Index (PSQI). No significant difference in the fractional representation of *Faecalibacterium* and sleep duration. Percent sleep duration and awakening events were found.	–	Increasing levels of *Faecalibacterium* in Bipolar patients can have positive psychiatric outcomes.
Lu et al. (2019)	Case‐control study	**Patients with BD: 36** BD I: 10 BD II: 26 Mean age: 32.64 years Education more than 12 years: 44.44% Mean BMI: 22.16 BMI > 25 = 25% Lost to follow‐up: 19 Males: 21 Females: 15 Drug Naive: 17 Medication free for 3 months.: 19 **Healthy controls‐27** Mean age: 28.89 years Education more than 12 years: 81.48% Mean BMI: 21.84 BMI > 25: 18.52% Males: 15 Females: 12	Counts of *Faeclibacterium prausnitzii, Bacteroides‐Prevotella, Eneterobactor spp*., and *Clostridium Cluster IV* were significantly increased in bipolar patients. microbial colonization resistance was significantly decreased in bipolar patients	–	–	Gut microbiota composition in patients with BD differed from that in HCs and was associated with illness severity and immune alterations. Expansion of the *Bacteroides–Prevotella* group and *Enterobacter* spp. indicated disturbance of gut microbiota. Decrease of Bifidobacteria to Enterobacteriaceae ratio was related to weakened microbial colonization resistance.
Lai et al. (2021)	Cross‐sectional study	Patients with BD‐25 and healthy controls (HC)‐28 Mean age of patients with BD: 36.9 Mean age of HC: 39.2 Females BD: 11 Males BD: 14 Females HC:15 Males HC:13 HAMD: 20.12 MDQ: 8.60 HAMA: 14.72 HCL‐32: 19.88 BPD‐I: 18 BPD‐II: 7 Atypical antipsychotics: 3 Anticonvulsants: 2 Lithium: 1 Antidepressants: 1 Combinations of the above medications: 15 Atypical antipsychotics + lithium: 2 Atypical antipsychotics + anticonvulsants: 1 Lithium +antidepressants: 1 Anticonvulsants + antidepressants: 6 Atypical antipsychotics + anticonvulsants + antidepressants: 2 Atypical antipsychotics + anticonvulsants + antidepressants lithium: 1	Decreased plasma Trp levels in BD	–	–	Gut microbiota changes can be used as biomarkers for BD identification; there are increased amounts of *Bacteroidetes, Firmicutes*, and *Actinobacteria* in patients with BD. There are impairments to the gut MiTBamp gene in patients with BD
Bengesser et al. (2019)	Cross‐sectional study	Patients with BD‐32 Mean age: 41.67 years Females with BD: 7 Males with BD: 25 BD depression: 13 Euthymia: 19 HAMD mean: 13 BDI mean: 14.73 YMRS mean: 0.81 BMI mean: 27.99	Methylation status (in %) of the *ARNTL* CpG position cg05733463 correlated significantly with gut bacterial diversity.	‐	–	There is a correlation between CpG methylation status of the clock gene ARNTL, gut microbiome diversity, and evenness in BD. Methylation status at cg05733463 of the clock gene *ARNTL* showed a negative correlation with bacterial diversity and evenness. Low microbiome diversity may lead to increased *ARNTL* and *MAOA* gene expression, leading to decreased breakdown and a promanic effect.
Reininghaus et al. (2020)	Cohort Study	Euthymic patients with BD ‐ 38 Mean age: 51.5 Females, %: 55 Males, %: 45 BMI: 30.1 YMRS:1.85 HAMD:1.75 Number of lifetime manic episodes: 9.28 Number of lifetime depressive episodes: 18.17 Duration of euthymia before testing, months: 6.89 Lithium, %: 40 Atypical antipsychotics, % : 40 Anticonvulsants, %: 20 SSRIs, %: 20 SNRI, %: 25 Tricyclics, %: 10	–	Improved cognition in euthymic patients with BD	*Lactobacillus, Bifidobacterium*, and *Lactococcus* supplementation improved cognitive function in patients with BD.	After 1 and 3 months of probiotic treatment, there was a significant improvement in attention, psychomotor processing speed, and executive function in bipolar patients.
Reininghaus et al. (2020)	Cohort Study	Euthymic patients with BD‐ 27 Mean age: 50.7 years Gender count: Females (%) 40.7 BMI mean‐29.0 Probiotic history (%)‐31.3 Nicotine dependence mean: 1.2 Psychiatric illness duration: 19.3 years Manic episodes: 9 Depressive episodes: 17.6 Current symptomatology: HAMD: 2.2 BDI: 25.0 YMRS: 2.4 MSS: 5.62 Lithium intake, % 40.7 SSRI (%)‐14.8 SNRI (%)‐33.3 Atypical antipsychotics, % 44.4 Anticonvulsants, % 25.9	–	Lowered manic symptoms, less ruminative thoughts.	*Lactobacillus* and *Bifidobacterium* variants in probiotic supplements improved manic symptoms	1 and 3 months of “OMNi‐BiOTiC” probiotic use showed a reduction in ruminative thoughts related to sad moods (possibly reducing the transition to depressive symptoms) as well as a significant reduction in manic symptoms.

*Note*. BDI‐II, Beck Depression Inventory; BMI, Body Mass Index; HAMD, Hamilton Depression Scale; HCL‐32, Hypomania; MADRS, Montgomery‐Åsberg Depression Rating Scale; MET‐minutes, metabolic equivalent minutes; MSS, Mania Symptom Scale; NOS, not otherwise specified; SNRIs, serotonin and noradrenaline reuptake inhibitor; SSRIs, Selective serotonin reuptake inhibitors; YMRS, Young Mania Rating Scale.

### 3.1 Gut–brain axis changes in BD

The following were observed in this analysis: (1) the composition of the gut microbiome; (2) the neurotransmitter pathway differences of gut microbiota; and (3) the gut microbiota composition and its relation to inflammation and serum lipids.

#### 3.1.1 Gut microbiota composition

There are significant differences in gut microbiota composition in patients with BD as compared to healthy controls (HCs) (Coello et al., 2019; Evans et al., 2017; Hu et al., 2019; Painold et al., 2019). *Flavonifractor, Actinobacteria*, and *Coriobacteriaceae* were found to be increased in patients with BD (Coello et al., 2019), while *Ruminococcus* and *Faecalibacterium* were found to be higher in HCs (Evans et al., 2017; Hu et al., 2019; Painold et al., 2019).

Coello et al. (2019) conducted a study that compared the gut microbiota composition between patients with BD, their first‐degree unaffected relatives, and HCs. A total of 64 genera were identified, with *Flavonifractor* being significantly higher in patients with BD (61%) compared to healthy individuals (39%, *p‐value*  =  6.3 × 10^−4^ *Q*  =  0.04). The prevalence of *Flavonifractor* in the first‐degree unaffected relatives (44%) differed from patients with BD (*Q*  =  0.02) but not from healthy individuals (*Q*  =  0.7).

An interesting finding of this study was that newly diagnosed patients with BD were 2.9 times (OR 2.9, 95%CI: 1.6−5.2, *p*  =  5.8 × 10^−4^, *Q*  =  0.04) more likely to have *Flavonifractor* detected compared to HCs. Despite adjusting for the presence of *Flavonifractor* in BD for age, sex, smoking, waist circumference, physical activity, and medication, BD was still associated with *Flavonifractor* prevalence. However, among patients with BD, smoking (OR: 3.0, 95%CI: 1.2−7.5, *p*  =  0.016) and female sex (OR: 2.4, 95%CI: 1.1−5.4, *p*  =  0.029) were associated with the presence of *Flavonifractor*.

Lu et al.’s results also found significant differences in the gut microbiota composition of patients with BD. They found that the counts of *Faecalibacterium prausnitzii*, *Bacteroides*–*Prevotella group, Atopobium Cluster, Enterobacter spp., and Clostridium Cluster IV* were significantly higher in the BD group compared with HCs (*p* = .030, *p* < .001, *p* < .001, *p* < .001, and *p* < .001, respectively) (Lu et al., 2019). The *Bacteroides*/*Enterobacter* (B/E) ratio of the BD group was significantly lower than the HCs (*p* = .001).

Notably, one study found differences in gut microbiota composition between patients with BD with and without depressive symptoms (Painold et al., 2019). In patients experiencing the depressive phase of BD, Enterobacteriaceae (Linear Discrimination Analysis [LDA] = 3.12, *p* = .044) were more abundant, while *Clostridiaceae* (LDA = 3.41, *p* = .048) and *Roseburia* (LDA = 3.13, *p* = .016) were more abundant in patients with BD with depressive symptom improvement. When comparing patients with BD and HCs, *Actinobacteria* (LDA = 4.82, *p* = .007) and *Coriobacteria* (LDA = 4.75, *p* = .010) were significantly more abundant in BD when compared with HCs, and *Ruminococcaceae* (LDA = 4.59, *p* = .018) and *Faecalibacterium* (LDA = 4.09, *p* = .039) were more abundant in HC when compared with BD (Painold et al., 2019).

Although most of the studies we examined found significant gut microbiota differences in patients with BD, there were some conflicting results noted in this analysis. Aizawa et al. (2019) noted that between patients with BD and controls, no significant differences were found in fecal *Bifidobacterium* (df = 1, 92; *F* = 0.34, *p* = .56, Partial η2 = 0.004) or fecal *Lactobacillus* counts (df = 1, 92; *F* = 0.14, *p* = .71, Partial η2 = 0.002). Male and female subjects were examined separately, and there were still no significant differences found for fecal bacterial counts. Separately comparing Bipolar I (*Bifidobacterium*: *df* = 1, 66; *F* = 0.05, *p* = .83, Partial η = 0.001 [Bipolar Disorder‐Fact Sheet, 2022]); (*Lactobacillus*: *df* = 1, 66; *F* = 0.46, *p* = .50, Partial η = 0.01 [Bipolar Disorder‐Fact Sheet, 2022]) and Bipolar II (*Bifidobacterium: df* = 1,79*; F =* 0.83*, p* = .36, Partial *η =* 0.01 [Bipolar Disorder‐Fact Sheet, 2022]); (*Lactobacillus* : *df* = 1, 79; *F* = 0.86, *p* = .36, Partial η = 0.01 [Bipolar Disorder‐Fact Sheet, 2022]) patients also yielded no significant differences for both fecal bacterial counts. BMI was also controlled for, with no change in results. Additionally, no significant partial correlation was found between bacterial counts and HAM‐D total score (for *Bifidobacterium*: ρ = −0.06, *p* = .72; for *Lactobacillus*: ρ = −0.24, *p* = .16) (Aizawa et al., 2019). Examination of bacterial counts and YMRS total score yielded no significant partial correlation as well (for *Bifidobacterium*: ρ = 0.11, *p* = .53; for *Lactobacillus*: ρ = 0.25, *p* = .14) (Aizawa et al., 2019). However, a significantly negative correlation was found between *Lactobacillus* counts and sleep (ρ = −0.45, *p* = .01 (Aizawa et al., 2019; Da Costa et al., 2016).

#### 3.1.2 Neurotransmitter pathway differences of gut microbiota

Lai et al. (2021) examined 25 bipolar patients and 28 HCs to compare the composition of gut microbiota and tryptophan (Trp) synthesis and metabolism‐related genes in patients with BD using SMS. The fecal samples of each group were analyzed using alpha‐diversity calculations via the Shannon, Fisher, and Simpson indexes. Using the Kyoto Encyclopedia of Genes and Genomes database (KEGG), there were two KEGG orthologies (KOs) significantly decreased and five significantly increased KOs that could affect the normal functioning of the Tryptophan pathway in patients with BD. One of the decreased KOs was K00837 (FDR.*p* < .001), aromatic aminotransferase, which is reported to cause less synthesis of tryptophan. Also, the KO1667 (FDR.*p* < .001), Tryptophanase enzyme, was found in higher abundance in BD patients.

Additionally, Painold et al. (2019) found that individuals with high Trp differed significantly in the genus *Lactobacillus* (LDA = 4.73, *p* < .001), the family of *Lactobacillaceae* (LDA = 4.73, *p* < .001), the family of *Coriobacteriaceae* (LDA = 4.41, *p* = .019) and *Clostridiaceae* (LDA = 3.68, *p* = .044) from individuals with low Trp (Painold et al., 2019).

#### 3.1.3 Gut microbiota composition and its relation to inflammation and serum lipids

Painold et al. (2019) investigated how gut microbiota composition in BD is related to depressive symptoms. Their analysis revealed that patients with BD with high IL‐6 levels showed significantly higher amounts of *Lactobacillus* (LDA = 4.43, *p* = .006) and *Streptococcus* (LDA = 3.75, *p* = .012) compared with BD individuals with lower IL‐6 (Perlis et al., 2006). Additionally, among patients with BD with higher cholesterol levels, there were significantly higher levels of *Clostridiaceae* (LDA = 3.48, *p* = .004) compared to patients with BD with low cholesterol levels.

#### 3.1.4 Genetic contributions to the gut microbiota

Bengesser et al.’s (2019) study was the only of its kind to investigate a genetic component of BD that contributes to altered gut microbiota levels. It was found that the methylation status (in %) of the Aryl Hydrocarbon Receptor Nuclear Translocator Like (ARNTL) CpG position g‐5733463 correlated significantly with gut bacterial diversity (Simpson index: *r* = −0.389, *p*  =  .0238) and evenness (Simpson evenness index: *r* = −0.358, *p*  =  .044) in individuals with BD. The alpha‐diversity, a count of the different bacterial taxa that exist in the gut and is measured by the Simpson index, differed significantly between patients with BD with current depressive symptoms (n  =  13) and those who were euthymic (n  =  19, F(1,30)  =  4.695, *p*  =   .039, Partial Eta2  =  0.144).

### 3.2 BD symptom changes after gut microbiota alterations

We analyzed studies that investigated how balancing gut microbiotas affected the severity of bipolar symptoms, such as mania and depressive symptoms, and found conflicting results.

Eslami Shahrbabaki et al. (2020) evaluated bipolar I patients after 8 weeks of probiotic use. Blind randomization methods were used to divide patients into a placebo and probiotic groups. Patients in both groups were able to receive lithium oxide (max: 900 mg/day), sodium valproate (max: 1200 mg/day), and risperidone if needed. The probiotic group received a probiotic capsule containing 1.8 × 109 CFU/capsule *Bifidobacterium bifidum, Bifidobacterium lactis, and Bifidobacterium langum*, and *Lactobacillus acidophilus*. The YMRS mania scale and Hamilton's depression scale questionnaires were completed by a psychiatry resident at the start of the study, before the intervention (probiotic capsule), at the 4‐week mark, and at the 8‐week mark. While both the probiotic and placebo groups displayed a decrease in mania and depression questionnaire scores throughout the duration of the study, it was found that patients in the probiotic group displayed significantly higher decreases in symptom severity via the Young Mania (*p* value = .001) and Hamilton (*p*‐value = .001) questionnaire scores throughout the three measured points of the study. However, even though the probiotic group had a more significant improvement in questionnaire scores throughout the 8‐week study as compared to the placebo group, there were no significant differences in overall mania (*p*‐value = .2) and depression (*p*‐value = .5) scores between placebo and probiotics patients with type 1 BD.

In their study, Reininghaus et al. (2020) examined how probiotic supplementation affected mood symptoms in euthymic patients with BD. All participants with BD were treated with probiotic supplements. There was no placebo group. Researchers instead compared manic symptom severity via the YMRS scale among the treatment group between different time points in the study as well as symptom improvement throughout the 3‐month duration of the study. They found that manic symptoms significantly decreased over time (all 3 months) (MSS: *F* (2, 18) = 3.621, *p* = .048; YMRS: *F* (2,17) = 4.751, *p* = .023). There was no significant improvement in symptoms between the different time points of the study

### 3.3 BD symptom changes after gut microbiota alterations and effectiveness of gut balancing methods

We analyzed studies that investigated how balancing gut microbiotas affected the severity of bipolar symptoms, such as mania and depressive symptoms, and found conflicting results. Other parameters, such as cognition, mood, and hospitalization, were investigated.

Eslami Shahrbabaki et al. (2020) evaluated bipolar I patients after 8 weeks of probiotic use. Blind randomization methods were used to divide patients into a placebo and probiotic groups. Patients in both groups were able to receive lithium oxide (max: 900 mg/day), sodium valproate (max: 1200 mg/day), and risperidone if needed. The probiotic group received a probiotic capsule containing 1.8 × 109 CFU/capsule *Bifidobacterium bifidum, Bifidobacterium lactis, and Bifidobacterium langum*, and *Lactobacillus acidophilus*. The YMRS mania scale and Hamilton's depression scale questionnaires were completed by a psychiatry resident at the start of the study, before the intervention (probiotic capsule), at the 4‐week mark, and at the 8‐week mark. While both the probiotic group and placebo group displayed a decrease in mania and depression questionnaire scores throughout the duration of the study, it was found that patients in the probiotic group displayed significantly higher decreases symptom severity via the Young Mania (*p* value = .001) and Hamilton (*p*‐value = .001) questionnaire scores throughout the three measured points of the study. However, even though the probiotic group had a more significant improvement in questionnaire scores throughout the 8‐week study as compared to the placebo group, there were no significant differences in overall mania (*p*‐value = .2) and depression (*p*‐value = .5) scores between placebo and probiotics patients with type 1 BD.

Dickerson et al. (2018) gave recently hospitalized manic patients *Lactobacillus rhamnosus* strain GG and *Bifidobacterium animalis* subsp. *Lactis* strain Bb12 over a a period of 24 weeks. During the study period, there were eight (24.2%) rehospitalizations (𝑥= 5.2, *p* = .022) (Bipolar disorder‐Fact Sheet, 2022) among the patients who received probiotics as compared to a total of 17 (51.5%) placebo group participants requiring at least one rehospitalization. Furthermore, the length of stay was significantly reduced in the probiotic group (2.8 days) in comparison to the placebo group (8.3 days) (𝑥 = 5.17, *p* = .17) (Bipolar Disorder‐Fact Sheet, 2022). Additionally, there were significant improvements in BPRS (*p* < .0001) and YMRS scales (repeated measures Analysis of Variance (ANOVA) by week, *F* = 10.84, *p* ← .0001).

Reininghaus et al. (2020) conducted two studies that investigated the effect of probiotic treatment on cognition among individuals with euthymic BD. In the first study (Reininghaus et al., 2020), they investigated how probiotic supplementation affected cognitive performance in euthymic bipolar patients and found a significant improvement of performance in attention and psychomotor processing speed after 1 and 3 months of treatment (*F* = 8.60; η2 = 0.49, *p* < .01). Furthermore, executive function measured with the TMT‐B, increased significantly over 3 months (*F* = 3.68; η2 = 0.29, *p* < .05). In the second study (Reininghaus et al., 2020), they investigated how probiotic supplementation affected psychological parameters associated with bipolar patients. All participants with BD were treated with *Lactobacillus* and *Bifidobacterium* probiotic supplements. There was no placebo group. Researchers instead compared manic symptom severity via the YMRS scale among the treatment groups between different time points in the study as well as symptom improvement throughout the 3‐month duration of the study. They found that manic symptoms significantly decreased over time (all 3 months) (MSS: *F* (2, 18) = 3.621, *p* = .048; YMRS: *F* (2,17) = 4.751, *p* = .023). There was no significant improvement in symptoms between the different time points of the study. However, it was found that, those who received probiotic supplementation with *Lactobacillus* and *Bifidobacterium* were found to have a significantly decreased Leiden Index of Depression Sensitivity‐Revised rumination score over time with three interval points: (*F* (2, 17) = 4.024, *p* = .037; *t*2 (mean 14.0 SD = 6.0) to *t*3(mean 11.9, SD = 5.6); F(1, 20) = 10.563, *p* = .004)).

## 4 DISCUSSION

The pathogenesis of BD remains unclear. However, it has been shown that gut microbiotas play an important role in its onset and progression (Aizawa et al., 2019; Bailey et al., 2011; Bengesser et al., 2019; Coello et al., 2019; Cryan & Dinan, 2012; Da Costa et al., 2016; Evans et al., 2017; Forsythe et al., 2010; Fountoulakis, 2012; Hu et al., 2019; Lai et al., 2021; Lu et al., 2019; Mayer, 2011; Misiak et al., 2020; Painold et al., 2019). The gut–brain axis is a system that has numerous pathways, and any change in the molecular components of this system can result in disease for the host (Appleton, 2018). Painold et al. (2019) investigated how gut microbiota composition in BD relates to disease states and found that *Lactobacillus* was found to have a direct correlation with higher tryptophan levels, essentially affecting neurotransmitter levels known to play a role in BD pathogenesis. This gut–brain axis connection between the gut microbiome and brain neurotransmitters illustrates how some bacteria can be used as “psychobiotics” (Painold et al., 2019). In addition to cytokines, neurotransmitter precursors like tryptophan have been found to be linked to patients with BD through microbiota alterations (Appleton, 2018; Forsythe et al., 2010; Lai et al., 2021; Lu et al., 2019; Misiak et al., 2020). Using shotgun metagenomics sequencing, Lai et al. (2021) conducted a study on 25 bipolar patients and 28 HCs to compare the composition of gut microbiota with tryptophan (Trp) synthesis and metabolism‐related genes in patients with BD. It was found that tryptophanase and aromatic aminotransferase enzyme changes caused reduced tryptophan synthesis and ultimately lower serotonin levels in bipolar patients. Alternatively, Bengesser et al. (2019) found that bipolar patients had increased methylation of the ARNTL gene, which functions to break down serotonin into its byproducts. The malfunctioning and ultimate inhibition of ARNTL in patients with BD contribute to the higher levels of serotonin seen in manic episodes.

The relationship between the gut–brain axis and neurotransmitter availability is further highlighted by associations found between gut microbiota composition and tryptophan metabolism (Painold et al., 2019; Valladares et al., 2013). Species such as *Streptococcus*, *Escherichia coli, Lactococcus, and Lactobacillus* were found in studies to make serotonin via Trp synthetase (O'mahony et al., 2015; Painold et al., 2019). Painold et al.’s (2019) study on microbiome differences among patients with bipolar depressive symptoms associated certain *Lactobacillus* species with increased tryptophan levels.

Reininghaus et al.’s study investigating probiotic use among patients with BD found that probiotic supplements consisting of *Lactobacillus and* Bifidobacterium were associated with lower scores of depressive and ruminative thoughts over the duration of the study, which agrees with prior studies highlighting a link between *Lactobacillus* and increased serotonin levels in patients with BD (Painold et al., 2019; Reininghaus et al., 2020; Valladares et al., 2013). However, Dickerson et al.’s (2018) study investigating hospitalization rates among patients with mania found that *Lactobacillus* and *Bifidobacterium* supplements were associated with fewer lower rates of rehospitalization, fewer days of hospitalization, and significantly improved YMRS mania and brief psychiatric rating scores. It is important to note, however, that Dickerson found a higher effect on the association of probiotic use and rehospitalization prevention among patients who had increased levels of inflammation at baseline. The conflicts between Reininghaus et al. and Dickerson et. al.’s study could be mediated by the fact that inflammation may be a key factor in BD pathogenesis and symptomology as highlighted by previous studies (Bailey et al., 2011; Borthakur et al., 2010; Dickerson et al., 2018; Dinan & Cryan, 2017; Mayer, 2011). Additionally, Dickerson et al. (2018), admit that their study could not have been able to capture symptom severity throughout the experimental period. As some of the participating patients had mixed BD, it is hard to tell whether the improvements noted by Dickerson et al. could have been due to the mediation of bipolar depressive symptoms. Nonetheless, these findings support the relationship that has been found between gut microbiota, altered tryptophan metabolism, inflammation, and the HPA axis (Painold et al., 2019). that should be explored further in future studies.

While gut microbiota aberrations were discovered in patients with BD when compared to HCs, the species attributed to BD symptoms were not consistent across studies. Painold et al. (2019), concluded from their study that *Faecalibacterium* could be a distinguishing feature between patients with BD and HCs. HCs displayed higher levels of the *Faecalibacterium* genus and the *Ruminococcaceae* family compared to HCs. Their findings correlated with Evans et al. (2017), whose study associated adequate levels of *Faecalibacterium* with better health outcomes among patients with BD. Aside from these similar findings, however, the associated microbiota compositions varied. Significant associations between higher levels of *Actinobacteria, Coriobacteriaceae, Faecalibacterium, Enterobacter, Flavonifractor, Clostridium*, and *Bacteroides* were found in patients with BD (Hu et al., 2019; Lu et al., 2019; Painold et al., 2019). Studies also found differences in microbiota diversity specific to bipolar symptoms and illness severity (Hu et al., 2019). BD patients experiencing depressive symptoms were found to have higher levels of *Enterobacteriaceae*, while *Clostridiaceae* and *Roseburia* were found to be more abundant in less symptomatic patients with BD (Evans et al., 2017; Hu et al., 2019). An important factor to note about the differences in the gut diversity among patients with BD is the fact that patients were taking psychiatric medication throughout the duration of these studies. It has been shown that psychiatric medication also affects gut microbiota composition (Painold et al., 2019), and the lack of standardization of treatment among participating patients could have influenced the results of these studies.

There were additional conflicting results in the literature. In another study that was conducted by Eslami Shahrbabaki et al. (2020), the use of *Bifidobacterium* and *Lactobacillus* probiotic formulas over an 8‐week period led to insignificant changes to manic and depressive symptoms between placebo and BD type 1 patients. This is in contrast to Reininghaus et al.’s (2020) study investigating probiotic use among patients with BD, which found that probiotic supplements consisting of *Lactobacillus and Bifidobacterium* were associated with significantly lower scores of depressive and ruminative thoughts over the duration of the study. However, an interesting factor to note is that the composition of specific genus species varied greatly, the only species both studies included in their probiotic supplement was *Bifidobacterium lactis*. It is possible that the inconsistencies found between these two studies were due to differences in the species of *Lactobacillus* and *Bifidobacterium* used. The differences in Reininghaus et al.’s and Shahrbabaki et al.’s results prove that the careful study of the exact genus, species, and subspecies affecting patients in BD is important in determining outcomes in reducing symptom severity.

There are two contraindicative pathways for the role the proinflammatory cytokine IL‐6 plays in BD. Painold et al. (2019) have found a direct correlation between increased IL‐6 levels and a higher *Lactobacillus, Bacilli*, and *Streptococcaceae* presence. In their study, *Lactobacillus* was linked to depressive symptom reduction in human subjects, thereby suggesting a higher IL‐6 concentration in patients with BD can reduce depression. However, in opposition to this, Getachew et al. (2018) have reported that reductions in IL‐6 concentrations can result in the cessation of depressive‐like behavior. They state that probiotics are known to have anti‐inflammatory effects, and this is done by reducing proinflammatory IL‐6 levels in the gut. A possible cause for the conflicting results could be confounding. Painold et al., note that patients with BD who had high BMIs had higher *Lactobacillus* than patients with BD who had lower BMIs, aligning with research that associates high amounts of *Lactobacilllus* with obesity (Million et al., 2013; Painold et al., 2019). Future studies would need to reconcile metabolic factors among patients with BD and gut microbiome aberrations.

Probiotics are not the only nonpharmaceuticals proven to have positive effects on BD. Other potential methods of treatment include vitamin D, ketamine, and charcoal (Cereda et al., 2021; Getachew et al., 2018; Wilkowska et al., 2020). Charcoal, a potent adsorbent, has been hypothesized to reduce not only systemic inflammation but manic symptoms in patients with BD 15 days after treatment initiation (Cereda et al., 2021). The use of Vitamin D has also been shown to reduce depressive and manic symptoms of bipolar patients (Cereda et al., 2021).

Wilkowska et al.’s (2020) study mentions evidence of ketamine playing a role in altering the pathogenesis of BD In their study, decreased *Lactobacillus* levels were associated with depression and the administration of ketamine in rat species has been found to reduce depressive‐like behaviors as well as suicide risk. Getachew et al. also found *Lactobacillus* to be increased in ketamine‐treated rats. Despite the positive outcomes reported in these initial studies, further research is needed as these treatment options are understudied.

## 5 LIMITATIONS

While our initial search yielded many results, only 12 were original experimental studies involving BD patients. Our cumulative sample size was 613 bipolar (BD) patients and 312 HCs. This is an ideal cumulative size, however, outside of one study (Evans et al., 2017), which had a sample size of 115 bipolar patients, and 64 control subjects, the other studies had relatively small sample sizes even before losing subjects to follow‐up. This left room for sampling error and left all but one of the articles included in this study with reduced design power. Additionally, most of these studies were of cohort, case‐control, or cross‐sectional design, preventing causal conclusions being drawn. Due to the different study designs, some potential biases could be present. Last, there were inconsistencies in the amount of potential confounding data collected per study, which could also make room for additional biases. While a few findings were replicated between studies, overall, they were very inconsistent. Despite these limitations, our study has demonstrated the need for further research regarding gut balancing methods and their impact on the treatment of BD.

## 6 CONCLUSIONS

In this systematic review, we aimed to assess how components of the gut–brain axis contribute to BD, to determine how balancing gut microbiotas affect the severity of symptoms, and to discuss and compare the efficacy of varying methods of balancing gut microbiotas in BD. There are significant associations between BD pathogenesis and alterations of gut microbiome diversity via immunomodulation, the HPA axis, and neurotransmitter alterations. Specific microbiome species could serve as therapeutic targets as adjunctive therapies for patients with BD to manage depressive and manic symptoms. Additionally, other gut‐balancing methods, such as ketamine, charcoal, and vitamin D supplementation, have also shown promise as adjunctive agents. Despite these promising findings, current studies investigating the gut–brain axis relationship among patients with BD are limited by small sample sizes and a lack of adequate replication of results. However, the conclusions drawn from this review highlight that there may be specific bacteria common to patients with BD that could serve as screening tools and therapeutic targets, of which future studies could investigate. We recommend that future studies place emphasis on the following:

1. Identify common microbiome alterations that can be linked to BD. The studies included in this review reported microbiome alterations among patients with BD, however, those alterations varied. There have yet to be additional studies that have replicated the findings of the articles mentioned.
2. Additional investigations into gut‐balancing methods aside from probiotics.
3. Further investigation of how gut microbiome alterations serve as epigenetic factors of the pathogenesis of BD. One of the included studies found a link between gut microbiota alterations and the epigenetic impact on the gene ARNTL, which is thought to play a role in BD pathogenesis (Bengesser et al., 2019). Additional studies that replicate this finding or find other possible epigenetic impacts would be impactful.

## DISCLAIMER

The products used for this research are commonly and predominantly used products in our area of research and country. There is absolutely no conflict of interest between the authors and producers of the products because we do not intend to use these products as an avenue for any litigation but for the advancement of knowledge. Also, the research was not funded by the producing company rather it was funded by the personal efforts of the authors.

## CONSENT

As per international standards or university standards, Participants’ written consent has been collected and preserved by the authors.

## CONFLICT OF INTEREST STATEMENT

The authors have declared that no competing interests exist.

### PEER REVIEW

The peer review history for this article is available at https://publons.com/publon/10.1002/brb3.3037

## Data Availability

Data sharing not applicable to this article as no datasets were generated or analyzed during the current study.
